# Impact of switching high-sensitivity cardiac troponin assays on risk stratification in suspected acute coronary syndrome

**DOI:** 10.1093/ehjacc/zuag042

**Published:** 2026-03-27

**Authors:** Ziwen Li, Jasper Boeddinghaus, Anda Bularga, Caelan Taggart, Ryan Wereski, Andrew R Chapman, Kuan Ken Lee, Dimitrios Doudesis, Christopher Tuck, Paul Fineran, Sara Jenks, Rebecca Pattenden, Jonathan Malo, Deepak Harry, Alexander J F Thurston, Yong Yong Tew, Michael McDermott, Alasdair Gray, Nicholas L M Cruden, Atul Anand, Nicholas L Mills

**Affiliations:** British Heart Foundation Centre of Research Excellence, University of Edinburgh, Chancellor′s Building, Edinburgh EH16 4SA, UK; British Heart Foundation Centre of Research Excellence, University of Edinburgh, Chancellor′s Building, Edinburgh EH16 4SA, UK; Cardiovascular Research Institute Basel (CRIB) and Department of Cardiology, University Hospital Basel, University of Basel, Basel, Switzerland; British Heart Foundation Centre of Research Excellence, University of Edinburgh, Chancellor′s Building, Edinburgh EH16 4SA, UK; British Heart Foundation Centre of Research Excellence, University of Edinburgh, Chancellor′s Building, Edinburgh EH16 4SA, UK; British Heart Foundation Centre of Research Excellence, University of Edinburgh, Chancellor′s Building, Edinburgh EH16 4SA, UK; British Heart Foundation Centre of Research Excellence, University of Edinburgh, Chancellor′s Building, Edinburgh EH16 4SA, UK; British Heart Foundation Centre of Research Excellence, University of Edinburgh, Chancellor′s Building, Edinburgh EH16 4SA, UK; British Heart Foundation Centre of Research Excellence, University of Edinburgh, Chancellor′s Building, Edinburgh EH16 4SA, UK; Usher Institute, The University of Edinburgh, Usher Building, 5-7 Little France Road, Edinburgh EH16 4UX, UK; British Heart Foundation Centre of Research Excellence, University of Edinburgh, Chancellor′s Building, Edinburgh EH16 4SA, UK; British Heart Foundation Centre of Research Excellence, University of Edinburgh, Chancellor′s Building, Edinburgh EH16 4SA, UK; Department Clinical Biochemistry, Royal Infirmary of Edinburgh, Edinburgh, UK; Department Clinical Biochemistry, Western General Hospital, Edinburgh, UK; Department Clinical Biochemistry, Royal Infirmary of Edinburgh, Edinburgh, UK; British Heart Foundation Centre of Research Excellence, University of Edinburgh, Chancellor′s Building, Edinburgh EH16 4SA, UK; British Heart Foundation Centre of Research Excellence, University of Edinburgh, Chancellor′s Building, Edinburgh EH16 4SA, UK; British Heart Foundation Centre of Research Excellence, University of Edinburgh, Chancellor′s Building, Edinburgh EH16 4SA, UK; British Heart Foundation Centre of Research Excellence, University of Edinburgh, Chancellor′s Building, Edinburgh EH16 4SA, UK; Department of Emergency Medicine, Royal Infirmary of Edinburgh, Edinburgh, UK; Usher Institute, The University of Edinburgh, Usher Building, 5-7 Little France Road, Edinburgh EH16 4UX, UK; Department of Cardiology, Edinburgh Heart Centre, Royal Infirmary of Edinburgh, Edinburgh, UK; British Heart Foundation Centre of Research Excellence, University of Edinburgh, Chancellor′s Building, Edinburgh EH16 4SA, UK; British Heart Foundation Centre of Research Excellence, University of Edinburgh, Chancellor′s Building, Edinburgh EH16 4SA, UK; Usher Institute, The University of Edinburgh, Usher Building, 5-7 Little France Road, Edinburgh EH16 4UX, UK

**Keywords:** Myocardial infarction, Cardiac troponin, Risk stratification

## Abstract

**Aims:**

Cardiac troponin is central to risk stratification in patients with suspected acute coronary syndrome. High-sensitivity assays measuring cardiac troponin (hs-cTn) I or T are recommended by international guidelines. However, diagnostic thresholds for these assays are derived from different populations. We aimed to evaluate whether the change in assay influenced risk stratification.

**Methods and results:**

This is a secondary analysis of the TWITCH-ED study enrolling 25 849 patients with suspected acute coronary syndrome between October 2020 and October 2022. In October 2021, sites changed from an hs-cTnI to an hs-cTnT assay. Study outcomes included discharge from the Emergency Department following a single cardiac troponin measurement, a composite outcome of subsequent myocardial infarction, heart failure hospitalization, or cardiovascular death at 1 year, and all-cause death at 1 year. Patients were stratified as low-risk at presentation using established thresholds. Higher proportions of patients were identified as low-risk (57.3% vs. 17.6%) and discharged following a single troponin measurement (47% vs. 30%) using the hs-cTnI assay, compared to the hs-cTnT assay (*P* < 0.001 for both). Those identified as low-risk with the hs-cTnT assay were younger (median 52 [inter-quartile range 40–62] vs. 41 [32–51] years) with fewer comorbidities, and had lower rates for the composite outcome (0.8% vs. 0.2%) and all-cause death (2.0% vs. 0.3%) (*P* < 0.001 for both) at 1 year.

**Conclusion:**

Risk stratification thresholds for hs-cTnI and hs-cTnT assays do not provide equivalent performance. The hs-cTnT assay and threshold identified fewer low-risk patients suitable for discharge, with fewer subsequent cardiovascular events at 1 year.

Key points

1. High-sensitivity cardiac troponin (hs-cTn) I and T assays and thresholds do not provide equivalent performance in risk stratification of patients with suspected acute coronary syndrome.
2. Lower proportions of patients were identified as low-risk and discharged following a single cardiac troponin measurement using the hs-cTnT assay and threshold.
3. Low-risk patients identified using the hs-cTnT assay and threshold were younger, with fewer comorbidities, and were less likely to have a subsequent cardiovascular event at 1 year.

## Introduction

High-sensitivity cardiac troponin (hs-cTn) is used in accelerated diagnostic pathways to risk-stratify patients with suspected acute coronary syndrome in the Emergency Department. International guidelines recommend the use of high-sensitivity assays measuring either cardiac troponin I or T, based on evidence of their comparable diagnostic performance.^1^ However, recent studies suggest cardiac troponin I and T may differ in the prediction of future adverse events. Moreover, the upper reference limits used to define myocardial injury and diagnose myocardial infarction are derived from different populations. Furthermore, the approach to defining thresholds that identify low-risk patients has differed between these assays with the limit of detection applied for hs-cTnT^2^ and a threshold based on clinical performance applied for hs-cTnI.^3^ This has resulted in uncertainty about whether hs-cTn I and T assays and their risk stratification thresholds are equivalent in clinical practice.

We previously conducted a prospective interrupted time series study (TWITCH-ED, NCT05748691) to evaluate the impact of switching from an hs-cTnI to an hs-cTnT assay on patient care and outcomes. The transition was associated with increased identification of myocardial injury, serial troponin measurements, and hospital admissions without an improvement in cardiovascular outcomes at 1 year.^4^ In this secondary analysis, we evaluated whether the change in assay influenced the effectiveness of risk stratification to identify low-risk patients at presentation suitable for discharge.

## Methods

In the TWITCH-ED study, 25 849 consecutive patients presenting with suspected acute coronary syndrome to three acute care hospitals in Scotland were identified between October 2020 and October 2022. All sites changed from the ARCHITECT*_STAT_* hs-cTnI (Abbott Diagnostics, USA) to the Gen 5 hs-cTnT (Roche Diagnostics, Switzerland) assay in October 2021. The study design has been reported previously.^4^ Throughout the study, patients were managed according to an established single-sample rule out pathway^5^ where care is guided by the initial 12-lead electrocardiogram and hs-cTn concentration for patients without ST-segment elevation. In this secondary analysis, outcomes included discharge from the Emergency Department following a single cardiac troponin measurement, a composite outcome of subsequent myocardial infarction, heart failure hospitalization, or cardiovascular death at 1 year, and all-cause death at 1 year. The study adhered to the principles of the Declaration of Helsinki and was approved by the Research Ethics Committee without requiring individual patient consent, as an evaluation of a change in routine care using approved tests.

All patients were stratified as low risk at presentation using the established risk stratification thresholds of <5 ng/L for both assays.^2,6^ Continuous variables were compared using the Wilcoxon rank sum test, and categorical variables using the Pearson’s *χ*^2^ test. Counts <5 were redacted for data protection. All statistical tests were two-sided with a 5% significance level. The study data are held in a Trusted Research Environment (DataLoch, Edinburgh, UK) accessible by approved individuals with the necessary information governance training. Summary data and the analysis code can be requested from the corresponding author.

## Results

**Figure 1:**
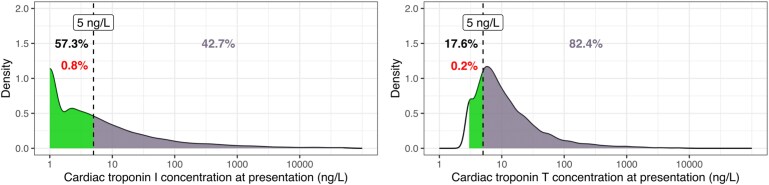
Density plots illustrating the proportion of patients stratified as low risk before and after transition from a high-sensitivity assay measuring cardiac troponin I to one measuring cardiac troponin T. Low risk (green) was defined by cardiac troponin concentrations below the risk stratification threshold of 5 ng/L at presentation using both the high-sensitivity cardiac troponin I and high-sensitivity cardiac troponin T assays in 13 146 and 12 703 consecutive patients, respectively. Event rate at 1 year for subsequent myocardial infarction, heart failure hospitalization, or cardiovascular death was highlighted in red for those stratified as low risk.

In 13 146 patients evaluated using the hs-cTnI assay, 57.3% (7534) had cardiac troponin I concentrations <5 ng/L at presentation and were identified as low risk. In contrast, of the 12 703 patients evaluated using the hs-cTnT assay after transition, 17.6% (2241) had cardiac troponin T concentrations <5 ng/L and were identified as low risk (*Figure 1*, *P* < 0.001). As such, following the transition to hs-cTnT testing, the proportion of patients discharged from the Emergency Department following a single cardiac troponin measurement reduced from 47% (6121/13 146) to 30% (3775/12 703) (*P* < 0.001).

Compared to patients identified as low risk with the hs-cTnI assay and threshold, those identified as low risk with the hs-cTnT assay and threshold were younger (median 52 [inter-quartile range 40–62] vs. 41 [32–51] years) and fewer had comorbidities, including previous myocardial infarction (5% vs. 1%) and diabetes mellitus (10% vs. 3%) (*P* < 0.001 for all). The event rate at 1 year was also lower in those identified as low risk using hs-cTnT for both subsequent myocardial infarction, heart failure hospitalization, or cardiovascular death (0.8 [57/7534] vs. 0.2 [<5/2241] %), and all cause death (2.0 [148/7534] vs. 0.3 [7/2241] %) (*P* < 0.001 for both).

## Discussion

Risk stratification thresholds for hs-cTn assays have been recommended by international practice guidelines based on evidence that they facilitate the safe discharge of low-risk patients at presentation.^7^ These thresholds are derived for accelerated diagnostic pathways to identify patients at low risk at presentation and are defined based on their ability to exclude myocardial infarction with a single measurement. For both the hs-cTnI and hs-cTnT assays evaluated here, the negative predictive value is >99.5% and sensitivity >97% for myocardial infarction or cardiac death at 30 days.^2,6^ However, few studies have compared the effectiveness of this approach between assays. Our findings demonstrated that the hs-cTnI assay and threshold used in this study identify three times as many patients as low risk at presentation. As a consequence, the proportion of patients discharged from the Emergency Department with a single measurement of cardiac troponin fell by a third following the implementation of hs-cTnT, explaining our prior observation that serial cardiac troponin testing and hospital admission increased following the transition to hs-cTnT. Furthermore, the characteristics of those classified as low risk differed significantly as did their risk of subsequent events.

It is worth noting that our findings on one specific hs-cTnI assay cannot be readily applied to other hs-cTnI assays, and we expect the performance of hs-cTnT assay to change with the upcoming Gen 6 hs-cTnT assay that has recently received CE marking.

In conclusion, risk stratification thresholds for hs-cTnI and hs-cTnT assays do not provide equivalent performance in the Emergency Department, with the hs-cTnT assay and threshold identifying fewer patients suitable for discharge. Whilst this contributed to additional testing, those identified as low risk by the hs-cTnT assay and threshold were less likely to have a subsequent cardiovascular event or death at 1 year.

## Data Availability

This study uses data provided by patients and collected by the NHS as part of their care and support. It has been facilitated by the DataLoch service (reference: DL_2022_046). Data may be accessed through DataLoch (dataloch.org) following successful application and approvals.
